# An intact membrane is essential for small extracellular vesicle‐induced modulation of α‐synuclein fibrillization

**DOI:** 10.1002/jev2.12034

**Published:** 2020-12-10

**Authors:** Cathryn L. Ugalde, Shane E. Gordon, Mitch Shambrook, Amirmohammad Nasiri Kenari, Bradley M. Coleman, Matthew A. Perugini, Victoria A. Lawson, David I. Finkelstein, Andrew F. Hill

**Affiliations:** ^1^ La Trobe Institute of Molecular Science La Trobe University Bundoora Victoria Australia; ^2^ Howard Florey Institute of Neuroscience and Mental Health Parkville Victoria Australia; ^3^ Department of Microbiology and Immunology University of Melbourne Parkville Victoria Australia; ^4^ Department of Biochemistry and Molecular Biology University of Melbourne Parkville Victoria Australia

**Keywords:** exosome, extracellular vesicle, neurodegeneration, protein misfolding, protein‐lipid interaction, α‐synuclein

## Abstract

The misfolding and fibrillization of the protein, α‐synuclein (αsyn), is associated with neurodegenerative disorders referred to as the synucleinopathies. Understanding the mechanisms of αsyn misfolding is an important area of interest given that αsyn misfolding contributes to disease pathogenesis. While many studies report the ability of synthetic lipid membranes to modulate αsyn folding, there is little data pertaining to the mechanism(s) of this interaction. αSyn has previously been shown to associate with small lipid vesicles released by cells called extracellular vesicles (EVs) and it is postulated these interactions may assist in the spreading of pathological forms of this protein. Together, this presents the need for robust characterisation studies on αsyn fibrillization using biologically‐derived vesicles. In this study, we comprehensively characterised the ability of lipid‐rich small extracellular vesicles (sEVs) to alter the misfolding of αsyn induced using the Protein Misfolding Cyclic Amplification (PMCA) assay. The biochemical and biophysical properties of misfolded αsyn were examined using a range of techniques including: Thioflavin T fluorescence, transmission electron microscopy, analytical centrifugation and western immunoblot coupled with protease resistance assays and soluble/insoluble fractionation. We show that sEVs cause an acceleration in αsyn fibrillization and provide comprehensive evidence that this results in an increase in the abundance of mature insoluble fibrillar species. In order to elucidate the relevance of the lipid membrane to this interaction, sEV lipid membranes were modified by treatment with methanol, or a combination of methanol and sarkosyl. These treatments altered the ultrastructure of the sEVs without changing the protein cargo. Critically, these modified sEVs had a reduced ability to influence αsyn fibrillization compared to untreated counterparts. This study reports the first comprehensive examination of αsyn:EV interactions and demonstrates that sEVs are powerful modulators of αsyn fibrillization, which is mediated by the sEV membrane. In doing so, this work provides strong evidence for a role of sEVs in contributing directly to αsyn misfolding in the synucleinopathy disorders.

## 1 INTRODUCTION

Neurodegenerative proteinopathies (NDPs) are a group of disorders where the aggregation of specific proteins in compartments of the central nervous system (CNS) occurs in conjugation with neuronal loss. The synucleinopathies are one of the most common NDP, which collectively include disorders associated with the intracellular accumulation of β‐sheet rich fibrils composed on misfolded α‐synuclein (αsyn). They include Parkinson's disease, dementia with Lewy Body and Multiple System Atrophy, among others. More than being a pathological hallmark of disease, evidence for αsyn being associated with the development of disease comes from the finding that mutations in its encoding gene, *SNCA*, cause early‐onset familial Parkinson's disease (Appel‐Cresswell et al., 2013; Kruger et al., 1998; Lesage et al., 2013; Polymeropoulos et al., 1997; Proukakis et al., 2013; Zarranz et al., 2004), as do duplications (Chartier‐Harlin et al., 2004; Ibáñez et al., 2004) or triplications (Singleton et al., 2003) of the gene. Likewise, there is now substantial evidence demonstrating αsyn adopts pathogenic properties upon misfolding that can contribute to disease pathogenesis (reviewed in: Ugalde, Finkelstein, Lawson, & Hill, 2016). Despite the strong link between familial mutations in *SNCA* and the aggregation of translated protein, the overwhelming majority of synucleinopathies are of sporadic origin. In these disorders, there is little understanding into the events that trigger or contribute to the misfolding of αsyn.

αSyn is a small, dynamic protein that is highly expressed in the presynapse (Jakes, Spillantini, & Goedert, 1994; Murphy, Rueter, Trojanowski, & Lee, 2000). While it is largely disordered, it has been reported to exist as a folded tetramer under certain native environments (Bartels, Choi, & Selkoe, 2011; Burré et al., 2013, Wang et al., 2011). Likewise, αsyn can associate with lipids whereby the interaction causes partial folding in the protein (Burré et al., 2013; Chandra, Chen, Rizo, Jahn, & Sudhof, 2003; Davidson, Jonas, Clayton, & George, 1998; Jao, Hegde, Chen, Haworth, & Langen, 2008). This αsyn:lipid interaction is considered to be relevant to the protein's normal biological function given that it has been shown to associate with synaptic vesicles (Lautenschläger et al., 2018) and be important for the assembly of the SNARE complex that is a protein complex that facilitates the docking of synaptic vesicles with the presynaptic membrane (Burré et al., 2010; Burré, Sharma, & Sudhof, 2014; Chandra, Gallardo, Fernandez‐Chacon, Schluter, & Sudhof, 2005; Choi et al., 2013). In contrast to roles in normal cellular functioning, under certain conditions αsyn:lipid interactions also appear to enhance the pathogenic properties of the protein. Many studies show preparations of synthetic vesicles or bilayers induce and/or accelerate protein folding (reviewed in: Ugalde, Lawson, Finkelstein, & Hill, 2019). However, while these studies are important to demonstrate this interaction may occur, caveats exist in the ability of biologically‐derived vesicles to be modelled using synthetic membranes that often do not harbour lipids in biologically‐relevant ratios or include peripheral and integral membrane proteins that foreseeably may be relevant co‐factors to αsyn:lipid interactions. Accordingly, further research is required using isolated vesicles to fully ascertain the role of lipids in disorders associated with the misfolding of αsyn.

Small (<200 nm) membranous vesicles of endosomal origin, small extracellar vesicles (sEVs) are released from most mammalian cell types. These lipid‐rich vesicles have been shown to accelerate αsyn fibrillization (Grey et al., 2015), however the mechanism by which this occurs or their effect on misfolded protein formed is unclear. This study utilized sEVs to scrutinize the role of biologically‐derived lipid vesicles in modulating αsyn fibrillization. Here, αsyn misfolding was studied using Protein Misfolding Cyclic Amplification (PMCA); an assay that was originally developed to study the misfolding of the prion protein, PrP^C^, into its misfolded isoform, PrP^Sc^ (Saborio, Permanne, & Soto, 2001) that is associated with another NDP, the prion disorders. The PMCA is an attractive tool to study αsyn misfolding because it has been shown to be capable of rapidly inducing misfolding in αsyn (Herva et al., 2014; Ugalde et al., 2020) and, given that a range of misfolded conformations present in human synucleinopathy disorders including oligomers and protofibrils (Garcia‐Esparcia et al., 2015; Kramer & Schulz‐Schaeffer 2007), the PMCA has advantages over traditional fibrillization techniques due to its ability to produce a heterogenous mix of various sized species (Herva et al., 2014; Ugalde et al., 2020). Using PMCA, we confirm sEVs alter αsyn misfolding and thoroughly characterise these changes using a range of assays: Thioflavin T (ThT), transmission electron microscopy (TEM), western immunoblot coupled with protease resistance assays and soluble/insoluble fractionation and analytical centrifugation. To our knowledge, this is the first time analytical centrifugation has been employed to report on a heterogenous population of misfolded protein in the presence of EVs and presents a powerful tool to accurately study protein:EV dynamics that requires little manipulation of the starting material. These studies confirmed sEVs accelerate αsyn misfolding and produces a larger quantity of large species. Next, in order to determine the role of the sEV membrane in exerting this effect, we disrupted the membrane ultrastructure of sEVs using combinations of methanol (MeOH) and sarkosyl. While these chemical‐modifications did not overtly change the protein profiles of the sEVs, their ability to alter misfolding was ablated. This is the first comprehensive study of αsyn:EV dynamics and provides strong evidence that sEVs are potent agents that can influence αsyn fibrillization in human synucleinopathy disorders.

## 2 MATERIALS AND METHODS

### 2.1 Preparation of recombinant protein for αsyn PMCA

Recombinant human αsyn was purchased from Monash Protein Production facility, Monash University Clayton VIC and prepared as described elsewhere (Ugalde et al., 2020). Briefly, protein was produced using wild‐type αsyn:pRSET B transformed into BL21 (DE3) Gold cells and amplified in luria broth (10 g/l bacterial tryptone (Merck, Kilsyth, VIC, Australia), 5 g/l yeast extract, 5 g/l NaCl, pH 7.5) containing ampicillin (100 μg/ml, Astral Scientific, Gymea, NSW, Australia). Protein was purified using a HiTrap Q column (GE Healthcare) and dialyzed against water with four buffer changes. Supplied protein was lyophilized and reconstituted in PBS+150 mM NaCl (PBSN) buffer. For reconstitution of protein, 10–16 mg protein was dissolved in 1 ml PBSN and centrifuged at 122,500 × *g* for 30 min, 4°C (Optima MAX Ultracentrifuge, Beckman Coulter) to sediment unwanted debris. Absorbance at 280 nm was obtained using a photometer (BioPhotometer, Eppendorf) and protein concentration determined by employing the Beer‐Lambert law. Protein was diluted to a final concentration of 90 μM and 60 μl aliquoted into PCR tubes and stored at −80°C until experimental use in αsyn PMCA.

### 2.2 αSyn PMCA

PMCA of αsyn, protease resistance studies and Thioflavin T experiments were performed as described previously (Ugalde et al., 2020). To determine the relative amounts of soluble and insoluble protein, ultracentrifugation was used. Following a PMCA reaction, protein was diluted in PBSN to fill a 200 μl capacity thick‐walled polyallomer tube (Beckman‐Coulter). Samples were spun at 436,000 × *g* for 1 h, room temperature, after which soluble proteins were collected as the supernatant. The pellet containing insoluble protein was washed in PBSN and centrifuged again under the same conditions. The supernatant was then discarded and the pellet resuspended in 8 M urea/PBSN and incubated for 30 min, room temperature. All samples were then diluted in 4× NuPAGE LDS sample buffer (Life Technologies) and boiled at 100°C for 10 min. Samples were resolved using SDS‐PAGE and western immunoblot employed to detect αsyn species using the antibody MJFR1 (abcam138501).

### 2.3 Coomassie brilliant blue staining of SDS‐PAGE gels

SDS‐PAGE gel containing resolved proteins was incubated with Coomassie brilliant blue stain (0.1% (w/v) Coomassie brilliant blue R‐250 (Sigma‐Aldrich) in 50% MeOH, 15% glacial acetic acid, 35% ddH_2_O (v/v)) overnight with constant agitation. Proteins were detected the next day after several washes in destain (20% EtOH, 10% glacial acetic acid, 70% dH_2_O(v/v)). Following destain, the gel was transferred to a white plastic insert and imaged using a developing dock (ChemiDoc MP Imaging System, Bio‐Rad).

### 2.4 Isolation of small EVs from GT1‐7 cells

Small EVs were harvested using differential ultracentrifugation from conditioned media of confluent GT1‐7 (RRID:CVCL_0281) cells (Vella et al., 2007). Briefly, cells were cultured to 50–70% confluency in complete media (OptiMEM supplemented with 10% (v/v) fetal calf serum (FCS), 1% GlutaMAX (v/v) and 1% penicillin/streptomycin 100 x (v/v); Life Technologies) before replacing media with EV‐depleted complete media for 48 h. Here, EV depletion of media was achieved by replacing FCS in complete media with the supernatant of FCS that was exposed to ultracentrifugation at 100,000 × *g* for a minimum of 18 h at 4°C. Upon harvesting conditioned media, cellular debris was removed by collecting the supernatant following centrifugation at 2000 × *g* for 10 min, room temperature, followed by ultracentrifugation at 10,000 × *g* for 30 min 4°C (Beckman Coulter Optima L‐100 XP Ultracentrifuge). Small EVs were then pelleted by ultracentrifugation at 100,000 × *g* for 1:10 h, 4°C (Beckman Coulter Optima L‐100 XP Ultracentrifuge). The supernatant was discarded and small EV‐enriched pellet washed by resuspension in a small volume of magnesium and calcium free PBS (dPBS), followed by a second ultracentrifugation at 100,000 × *g* as per the conditions above. The supernatant was aspirated and pellet resuspended in dPBS. Concurrent to all sEV isolations, the parental cells were harvested and pelleted by centrifugation at 700 × *g* for 3 min. Cells were washed in warm dPBS, pelleted by centrifugation and resuspended in dPBS.

### 2.5 Preparation of cells and sEVs added to αsyn PMCA

For studies using sEVs and cell extracts, a known dilution was taken from the stock (unlysed and diluted in dPBS) and lysis performed in buffer (150 mM NaCl, 50 mM Tris pH 7.4, 1% (v/v) Triton X‐100, 1% (w/v) sodium deoxycholate (Sigma‐Aldrich) and cOmplete ULTRA protease inhibitor in ddH_2_O) on ice for 20 min. Samples were centrifuged at 16,000 × *g* for 10 min (4°C) and protein concentration of the supernatant determined using the bicinchonic acid assay (Thermo Fisher Scientific). Concentrations of stock samples were obtained by accounting for the dilution of the sample in lysis buffer. Cells and sEVs were then diluted in dPBS to a concentration of 3.25 μg/μl and 5 μl added to αsyn aliquots (for a final concentration of 0.25 μg/μl) and stored at −80°C until experimental use.

### 2.6 Chemical modification of sEVs with methanol and sarkosyl

Chemical modification of sEVs was performed on triplicate sets that had been thawed on ice and diluted in cold dPBS to 3.25 μg/μl in a final volume of 40 μl. Each set was designated as being solubilized with methanol or both methanol and sarkosyl (Set 1 and Set 2, respectively), or the untreated control (Set 3). Here, sarkosyl (4% (w/v)) was added to Set 2 at a volume ratio of 1:1, while the equivalent volume of dPBS was added to Set 1 and 3. Tubes were incubated on ice for 30 min. Ice‐cold methanol was then added to Set 1 and 2 (4× volume, 320 μl), before being briefly vortexed. All sets were stored at −20°C overnight. Samples were pelleted by centrifugation at 16,100 × *g* for 30 min, 4°C to pellet samples. For Set 1 and Set 2: the supernatant was aspirated, pellet washed in 500 ml methanol and centrifuged again as per the above parameters. Supernatants were aspirated a final time and pellets air‐dried for 30 min, room temperature. During the centrifugation steps, Set 3 followed the same protocol except dPBS was substituted as the wash buffer. All sets were then resuspended in 40 μl dPBS and stored at −80°C. Before use in αsyn PMCA, thawed samples were gently resuspended using low power water bath sonicator at 4°C (Soniclean; 2× rounds of 20 s sonications, separated by 30 s rest on ice).

### 2.7 Transmission electron microscopy

For imaging sEVs and PMCA products using transmission electron microscopy (TEM), samples were applied to formvar‐coated grids with heavy carbon coating after fixation in glutaraldehyde (2% v/w in H_2_O) for 30 mins, room temperature, or overnight at 4°C. Samples were visualized by negative staining with uranyl acetate (2% w/v in H_2_O), with images captured using a Joel JEM‐2100 electron microscope.

### 2.8 Nanoparticle tracking analysis of isolated EVs using Zetaview

Small EVs were analysed for size and concentration via Zetaview installed with a 405 nm laser diode (Particle Metrix, PMX‐120). Vesicles were prepared with a 1:10,000 dilution in dPBS and recorded with an average of 223 particles per frame. For each cycle, 11 positions were taken with the following analysis parameters: camera sensitivity: 80, max area: 1000, min area: 5, min brightness: 30, min tracelength: 15. dPBS was analysed for background.

### 2.9 Analytical centrifugation

Sedimentation velocity experiments using PMCA products were performed using a Beckman Coulter XL‐A analytical ultracentrifuge equipped with absorbance optics and an An50 Ti 8‐hole rotor using similar methods as described previously (Atkinson et al., 2012; Burgess et al., 2008; Gupta, Soares da Costa, Faou, Dogovski, & Perugini, 2018; Peverelli, Soares da Costa, Kirby, & Perugini, 2016). Briefly, 2‐channel epon centrepiece quartz cells were loaded with 300–380 μl of α‐synuclein (initial protein concentration of 14 μM) and 320–400 μl of reference buffer (2.7 mM potassium chloride, 10 mM phosphate buffer, 0.29 M sodium chloride, pH 7.3–7.5). Data (up to 200 scans) were collected at 37°C continuously in absorbance mode using a wavelength of either 230 nm or 233 nm, rotor speed of 10 000 rpm, radial range of 6.0–7.3 cm, and radial step size of 0.003 cm without averaging. Multiple time‐staggered sedimentation velocity scans were analysed with the enhanced van Holde‐Weischet (vHW) method using the software UltraScan3 v4.0 (release 5699: http://www.ultrascan.aucsolutions.com/) (Demeler & van Holde, 2004) and apparent sedimentation coefficient distributions reported.

## 3 RESULTS

### 3.1 Characterization of PMCA‐generated misfolded αsyn

**FIGURE 1:**
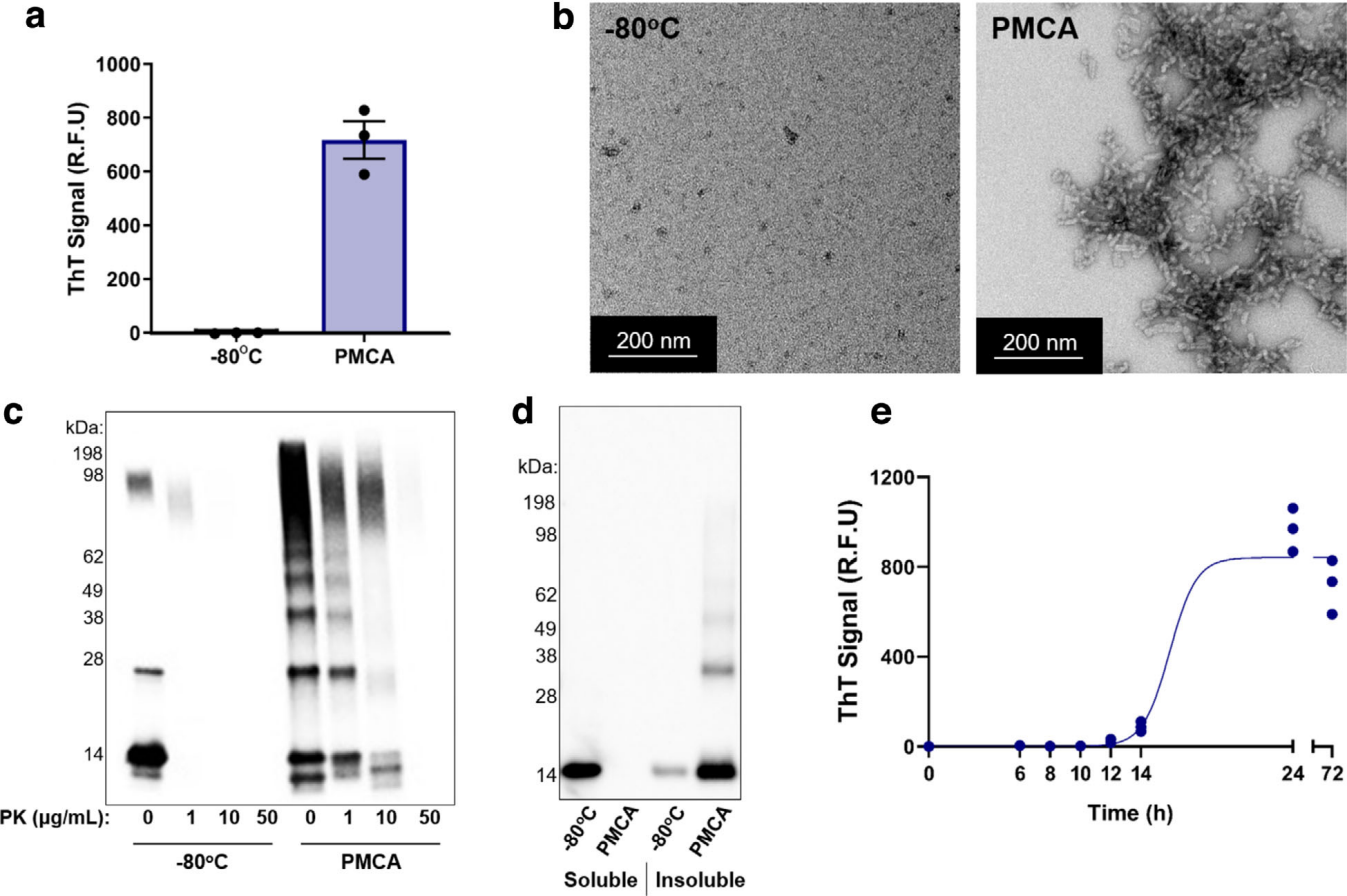
PMCA inducing misfolding of αsyn. 90 μM αsyn protein exposed to PMCA for 72 h was characterized by: (a) ThT fluorescence where the values obtained for each experimental replicate represented the average fluorescence of triplicate wells after subtraction of a blank well to account for background fluorescence (R.F.U = relative fluorescent units). Data presented as mean ± SEM (*n* = 3). A sample left at −80°C served as the monomeric protein control. (b) Transmission electron microscopy (TEM) of PMCA generated αsyn or −80°C monomeric proteins, (c) Western immunoblot analysis using αsyn‐specific monoclonal antibody MJFR1 (amino acid specificity: 118–123) in the absence and presence of proteinase K, or (d) following ultracentrifugation to separate into soluble and insoluble fractions. (e) ThT was also used to study the kinetics of αsyn fibrillization that showed the sigmoidal curve fit of β‐sheet amplification plateaued after 24 h exposure to PMCA. Data collection and analysis are as per (a), *n* = 3

The ability of PMCA to induce misfolding in αsyn was first confirmed following a previously published method that involved exposing human recombinant protein (90 μM) to PMCA for a total process time of 72 h (Ugalde et al., 2020). Misfolded αsyn protein was characterized using standard techniques routinely used to detect misfolded protein: Thioflavin T (ThT) fluorescence, western immunoblot (protease resistance and soluble/insoluble fractionation) and TEM. ThT is a dye that exhibits a fluorescence shift upon binding to β‐sheet structures and therefore is universally used to detect mature fibrils (Biancalana & Koide 2010; Vassar & Culling, 1959). Upon exposure to PMCA for 72 h, αsyn exhibited strong ThT reactivity, while an equivalent sample that was stored at −80°C for the duration of the PMCA process (to serve as the non‐PMCA control) displayed no fluorescent signal (Figure 1a; −80°C vs. PMCA; −1.3 ± 1.0 and 717.2 ± 69.4 relative fluorescent units; R.F.U.). Complementary to the ThT data, the generation of mature αsyn fibrils using PMCA was further confirmed using TEM. Using TEM, protein exposed to PMCA was found to form rod‐like fibrils (Figure 1b) consistent with the morphology of fibrils produced using PMCA reported by others (Herva et al., 2014; Ugalde et al., 2020). No fibrils were observed in the non‐PMCA, −80°C sample (Figure 1b). Western immunoblot was next employed to further characterise the misfolded protein. Because PMCA‐generated misfolded αsyn exhibits enhanced resistance to protease digestion compared to monomeric protein (Herva et al., 2014), SDS‐PAGE and western immunoblot coupled with proteinase K (PK) digestion can be used to detect misfolded conformations. The resistance of PMCA products to PK was determined by exposing samples to 0, 1, 10 or 50 μg/ml PK prior to western immunoblot. Results showed that PMCA‐generated protein is partially resistant to PK, with immunoreactivity persisting in the PMCA samples at all concentrations of PK tested – up to 50 μg/ml PK. As expected, the abundance of protein detected was dependent on the concentration of PK. In contrast, the −80°C monomeric αsyn was almost entirely digested in 1 μg/ml PK, and no protein was found upon treatment with 10 or 50 μg/ml PK (Figure 1c). Complementary to studies on PK resistance, PMCA‐generated αsyn or −80°C equivalents were exposed to ultracentrifugation to separate out the soluble and insoluble species. Exposing the pellet containing insoluble protein with 8 M urea followed by SDS‐PAGE analysis allowed detection of insoluble proteins. Upon performing this technique with −80°C and PMCA‐exposed protein, the soluble fraction showed strong immunoreactivity for −80°C‐exposed protein at the molecular weight of the monomer (14 kDa) which was absent in the PMCA‐treated sample (Figure 1d). In contrast, the PMCA sample contained the most αsyn protein in the insoluble fraction (Figure 1d). Collectively, these studies confirm the generation of misfolded protein and show the utility of ThT, TEM and western immunoblot to detect misfolded conformations.

Measuring time‐dependent rises in ThT‐reactivity is also used to measure the kinetics of protein misfolding that follows a sigmoidal path of fibrillization (Gade Malmos et al., 2017). In order to optimise this assay for PMCA‐generated αsyn misfolding, a time‐course experiment was performed. Here, ThT fluorescence was measured in protein that was added sequentially to PMCA such that the total time in PMCA ranged from 0 to 72 h. Results showed small increases in ThT reactivity that were first observed at 12 h (Figure 1e) and these values increased time‐dependently thereafter until 24 h. After this time, ThT remained stable (24 h vs. 72 h; 966.4 ± 55.8 and 717.2 ± 69.4 R.F.U.). This confirmed measuring ThT reactivity in αsyn exposed to PMCA is time dependent and can be used as a tool to measure the rate of fibrillization. Given that the plateau of the curve was observed beyond 24 h, we concluded that quantifying the rate of αsyn misfolding using this assay may be performed using a 24 h PMCA format.

### 3.2 Small EVs (sEVs) accelerate PMCA‐induced αsyn fibrillization

**FIGURE 2:**
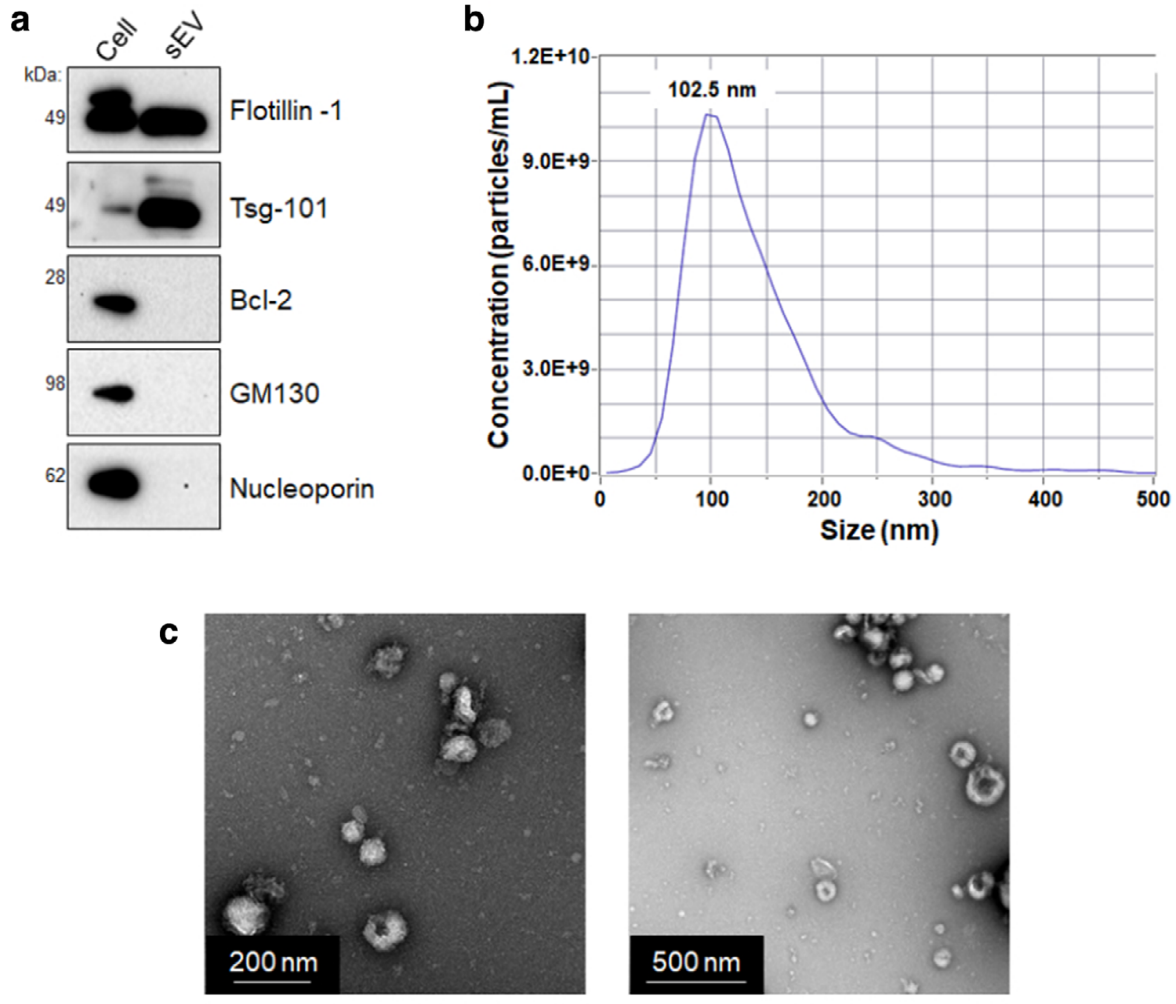
Characterization of small extracellular vesicles (sEVs) isolated from GT1‐7 conditioned media. Small EVs were isolated from conditioned cell culture media of GT1‐7 cells using differential ultracentrifugation. Characterization of sEVs was performed by: (a) Western immunoblot to detect the presence of EV markers (flotillin‐1 and tsg‐101) and absence of markers indicating contaminating from cellular compartments (Bcl‐2, GM130, Nucleoporin) in sEVs compared to lysate from the parental GT1‐7 cells, (b) ZetaView analysis, where the mode particle size detected was 102.5 nm and (c) TEM

Having established tools to assess PMCA‐generated αsyn, the effect of sEVs in this system was next studied. Small EVs used this study were isolated from the conditioned media of confluent GT1‐7 cells. This is an immortalized mouse hypothalamic cell line in which the content of their isolated EVs have been extensively characterized (Bellingham, Coleman, & Hill, 2012; Coleman, Hanssen, Lawson, & Hill, 2012; Quek et al., 2017). Small EV characterization was performed using western immunoblot on lysed samples to show that compared to lysate derived from the parental cell line, sEVs were enriched in flotillin‐1 and tumour susceptibility gene‐101 (tsg‐101). By comparison, markers of mitochondria, Golgi apparatus and the nucleus (Bcl‐2, GM130 and nucleoporin, respectively) were absent in sEVs but present in the cell extract (Figure 2a). The differential profile of these markers confirms harvested vesicles are of endosomal origin and have no apparent cytosolic contamination. The size distribution of vesicles harvested was examined next using ZetaView tracking analysis. Here, the histogram reported the mode vesicle size of the population to be 102.5 nm (Figure 2b). TEM was used to show the presence of vesicles < 200 nm that had the morphological features of GT1‐7‐derived sEVs (Bellingham et al., 2012; Coleman et al., 2012; Quek et al., 2017) (Figure 2c). These experiments meet the minimum characterization information required for sEVs as outlined in the most recent position statement from the International Society of Extracellular Vesicles (Théry et al., 2018).

**FIGURE 3:**
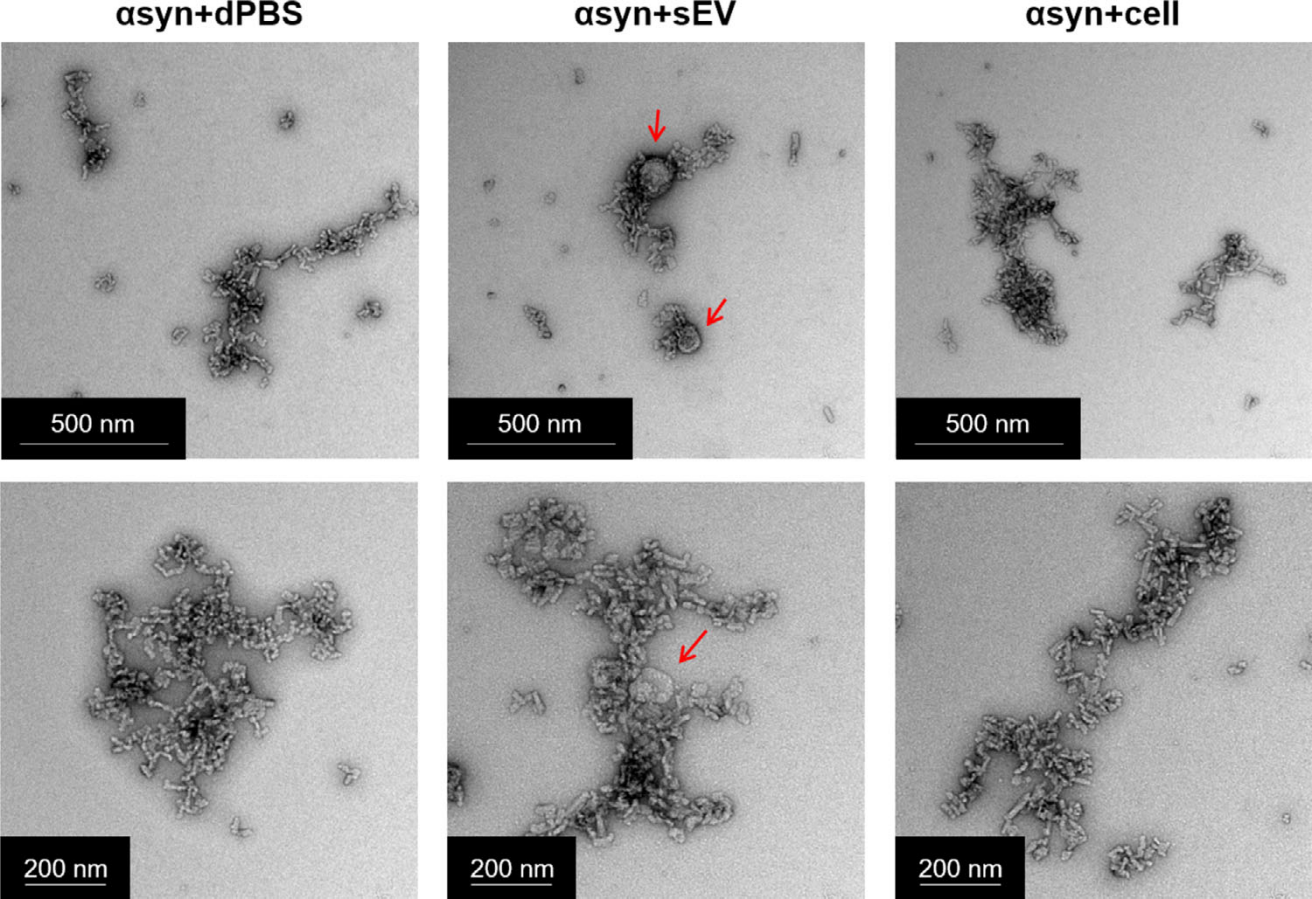
Transmission electron microscopy (TEM) images of αsyn fibrils formed in the presence of small EVs or cells. The morphology of misfolded αsyn formed in the presence of sEVs, cells (cell) or buffer only (dPBS) was assessed using TEM. Red arrows indicate the detection of intact sEVs following PMCA

To study the effect of sEVs on αsyn fibrillization they were added to αsyn and exposed to PMCA or left at −80°C. Fibrils formed were compared to αsyn samples containing the equivalent volume and protein concentration of cells, or buffer only (dPBS). We first used TEM to provide a global view of the morphology of αsyn species produced in the presence of sEVs or cells. Due to the highly heterogenous distribution of sizes, the TEM could not distinguish clearly any overt differences in the morphology or size of αsyn species formed between the treatment groups (Figure 3). However, an important observation using TEM was made in identifying the presence of intact sEVs in the αsyn+sEV sample (indicated by red arrows). This information demonstrates that a proportion, if not all sEVs remain intact during the fibrillization process and is evidence for changes seen in the sEV treatment group compared to cell or dPBS groups being attributable to interactions αsyn has with the membrane of the sEV.

**FIGURE 4:**
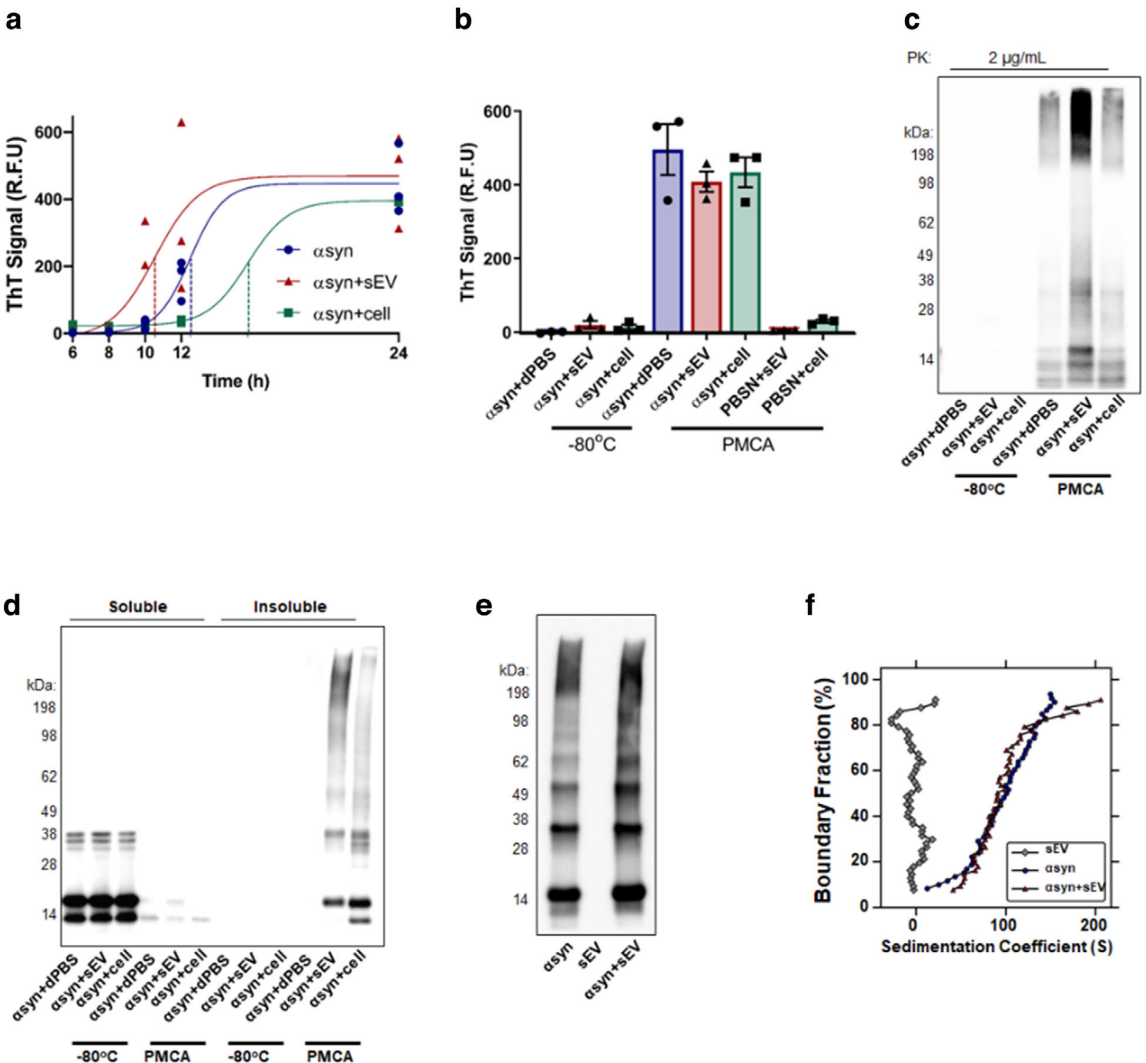
Small EVs accelerate αsyn fibrillization. (a‐b) Equivalent volume and protein amounts of sEVs and cells (cell), or the buffer they were diluted in (dPBS), were added to αsyn and exposed to PMCA. (a) A 24 h PMCA format was used to assess the kinetics of αsyn fibrillization in the presence of sEV, cell or dPBS. Changes to rates of fibrillization was determined by measuring ThT in samples exposed to PMCA for 6, 8, 10, 12 and 24 hours and a sigmoidal line fitted to the data. The t1/2 of αsyn, αsyn+sEV and αsyn+cell was 12.5, 10.55 and 15.67 h, respectively. Values obtained for each experimental replicate represented the average fluorescence of triplicate wells after subtraction of a blank well to account for background fluorescence (R.F.U = relative fluorescent units). Data presented as mean (*n* = 3). (b) PMCA was used to assess ThT fluorescence in 72 h PMCA misfolded αsyn protein. Additional samples containing sEV or cell void of αsyn exposed to PMCA (72 h) and equivalent αsyn+sEV or cell samples not exposed to PMCA (0 h, −80°C) served to confirm no inherent fluorescence was contributed by the biological source. Data collection and analysis are as per (a), *n* = 3. Following a 72 h PMCA, the properties of fibrils formed were further examined by (c) PK digestion to detect PK resistant product following exposure of samples to 2 μg/ml PK or (d) soluble/insoluble fractionation prior to western immunoblot using αsyn‐specific monoclonal antibody MJFR1 (amino acid specificity: 118–123). (e) To ensure no inherent αsyn reactivity was contributed by sEVs, western immunoblot was performed on αsyn only, sEV only or αsyn+sEV samples exposed to 72 h PMCA. (f) Enhanced van Holde‐Weischet integral distributions from extrapolation of analytical centrifugation sedimentation velocity data shown in Fig S1A‐C. Apparent sedimentation coefficient distributions are shown as a function of boundary fraction for sEVs alone (grey diamonds) and αsyn prepared in the absence (blue circles) or presence of sEVs (red upright triangles)

Given that sEVs have been shown to accelerate αsyn misfolding using traditional techniques of fibrillization (Grey et al., 2015), we first sought to determine whether this effect was also seen using PMCA as the method of promoting αsyn fibrillization. Following the optimisation of misfolding kinetics in Figure 1e, ThT was measured at multiple timepoints over 24 h to study the kinetics of αsyn misfolding in the presence of sEVs, compared to dPBS or cell extract (Figure 4a). Results showed that sEVs accelerated αsyn misfolding that was first detected by elevations in ThT at 10 h (αsyn+dPBS, αsyn+sEV and αsyn+cell: 28.4±8.8, 188.4 ± 90.3 and 25.6 ± 1.7 R.F.U., respectively) and 12 h (αsyn+dPBS, αsyn+sEV and αsyn+cell: 164.7±35.0, 347.7 ± 147.4 and 36 ± 2.5 R.F.U., respectively). Consistent with the sigmoidal fit of ThT data, these observed differences between groups were lost after exposure to PMCA for 24 h (αsyn+dPBS, αsyn+sEV and αsyn+cell: 447 ± 61.3, 472.7 ± 81.6 and 395.6 ± 2.4 R.F.U., respectively). Following plotting of the sigmoidal curve for each sample, the t1/2 time was calculated as 12.5, 10.55 and 15.67 h for αsyn+dPBS, αsyn+sEV and αsyn+cell, respectively. This finding shows that sEVs accelerate αsyn misfolding and given that cells do not facilitate αsyn misfolding, this confirms the effects of sEVs are not a result of general molecule crowding compared to the buffer control.

An additional important experiment to complement the ThT data detailed in Figure 4a is to confirm that no inherent ThT fluorescence is contributed by the sEVs or cells. To address this, ThT was measured in samples exposed to PMCA or not (−80°C), in samples that both contained αsyn or were void of protein. Here, minimal ThT reactivity was observed in all −80°C samples (αsyn+dPBS, αsyn+sEV and αsyn+cell: −0.2±1.6, 19.6±10.6 and 13.1±7.5 R.F.U, respectively) as well as αsyn‐free PBSN samples containing sEVs or cells which underwent PMCA (Figure 4b; PBSN+sEV vs. PBSN+cell: 5.6 ± 0.3 and 29.8 ± 4.40 R.F.U, respectively). As expected, ThT reactivity was observed in samples containing αsyn exposed to PMCA for 72 h (Figure 4b; αsyn+dPBS, αsyn+sEV and αsyn+cell: 495.8±69.2, 408.3±27.8 and 433.9±40.4 R.F.U, respectively). Consistent with Figure 1e and reported by others (Grey et al., 2015), no differences were seen between the groups at 72 h due to the plateauing effect of αsyn‐induced rises in ThT‐signal over time. Based on these observations, it can be concluded that the ThT data obtained from this experimental setup are a reliable measure of αsyn misfolding.

In order to determine what effect sEVs have on the misfolded protein formed, we next employed protease resistance experiments using samples exposed to PMCA for 72 h. Here, assessment on the resistance of species formed to digestion with 2 μg/ml PK revealed sEVs increased the amount of PK resistant species compared to dPBS or cells (Figure 4c), while the effective digestion of protein in −80°C samples confirmed that sEVs do not interfere with the ability of PK to digest protein. The load of soluble and insoluble protein was next assessed in these samples (Figure 4d). No differences were found in the soluble monomeric protein between groups that were not exposed to PMCA (−80°C), and all lacked insoluble content (Figure 4d). Importantly, PMCA‐generated protein formed in the presence of sEVs had more insoluble content compared to buffer or cell‐treated counterparts. An important consideration to using western immunoblot to detect misfolded protein is potential contamination from sEV‐associated αsyn given that αsyn has been found within sEVs (Emmanouilidou et al., 2010). To enable discrimination between EV‐associated αsyn and the recombinant protein misfolded using immunodetection, the sEVs and recombinant αsyn used in the current study were from different species (mouse and human, respectively) and the αsyn antibody used in these experiments (MJFR1) is not reported to react with mouse αsyn. However, we cannot exclude the possibility of weak contamination due to the antibody detecting mouse αsyn. To this end, αsyn immunoreactivity was tested in PMCA products formed from αsyn alone, αsyn+sEVS or sEVs void of any recombinant protein. Shown in Figure 3e, no signal was observed in sEVs alone. Collectively, these data confirm no inherent reactivity from western immunoblot is being contributed by αsyn content within sEVs and hence confirms sEVs increase the abundance of mature fibrils.

We additionally examined the effects of sEVs on PMCA‐mediated αsyn fibrillization in vitro using sedimentation velocity analytical centrifugation as described previously (Ugalde et al., 2020). To provide a robust assessment of composition differences between samples, raw absorbance‐detected sedimentation profiles (Figure S1A‐C) were analysed using the model‐independent enhanced van Holde‐Weischet (vHW) method (Demeler & van Holde 2004). As shown in Figure 4f, αsyn in the absence of sEVs yielded a broad distribution spanning 0 to 150 S, indicating a complex mixture of components with varying sedimentation coefficients. The addition of sEVs to αsyn resulted in a marked right shift in the distribution at higher boundary fractions (up to ∼210 S), reflecting a trend towards faster sedimenting (i.e. higher *s* value) species. Contrastingly, sedimentation velocity data for sEVs in the absence of αsyn yielded a significantly narrower sedimentation coefficient distribution up to only ∼25 S. Thus, these data suggest that changes in sedimentation coefficient distributions for αsyn observed in the presence of sEVs are unlikely to result directly from sedimentation of the sEV particles, and hence, support other data reporting an increase in the abundance of large fibrillar species produced by αsyn in the presence of sEVs.

### 3.3 Chemical modification disrupts the lipid membrane of sEVs

**FIGURE 5:**
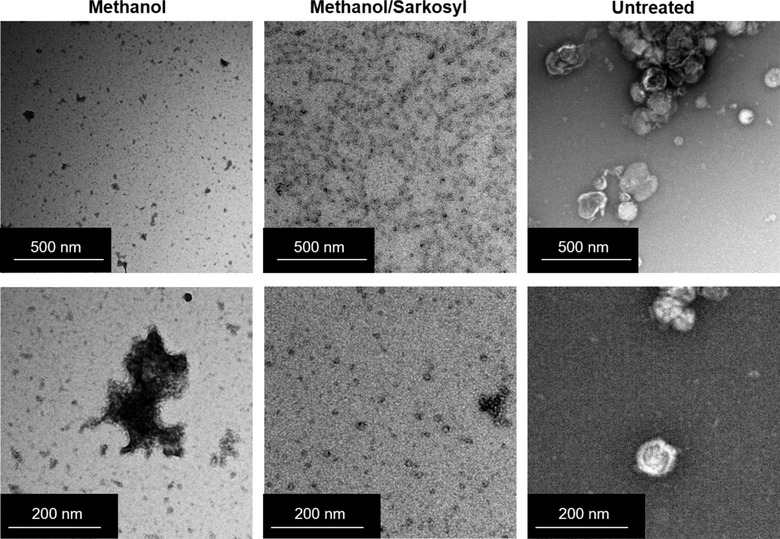
Transmission electron microscopy (TEM) images of small EVs following lipid solubilisation with methanol and sarkosyl. Chemical modification of whole sEVs was performed by overnight methanol precipitation alone or in combination with sarkosyl treatment. An additional group was used as a control for solubilisation (untreated) and instead was stored in dPBS during the precipitation process

The finding that intact sEVs are present following PMCA suggests that the lipid membrane composition or structure is what drives their observed effects on αsyn misfolding. To test whether this may be true, sEVs were chemically modified using sarkosyl and methanol, or methanol alone. The treatment of such chemicals to sEVs causes alterations to the ultrastructure of the vesicle and their lipid composition: methanol and sarkosyl disrupt lipid bilayers, making them more fluid and permeable associated with lateral diffusion of lipids (Anwar, Brown, Britten, & Lambert, 1983; Patra et al., 2006; Pinisetty, Moldovan, & Devireddy, 2006). The consequence of these chemical modifications to the morphology of sEVs was confirmed using TEM. Shown in Figure 5, both methanol and methanol/sarkosyl treatments caused dramatic changes to the ultrastructure of sEVs: methanol treatment produced largely random protein aggregates and sEVs with distorted membrane structures, while the addition of sarkosyl with methanol caused the generation of small micelles. In comparison, untreated sEVs remained morphologically intact throughout the treatment process (Figure 5).

### 3.4 An intact sEV membrane is required for its ability to alter αsyn fibrillization

**FIGURE 6:**
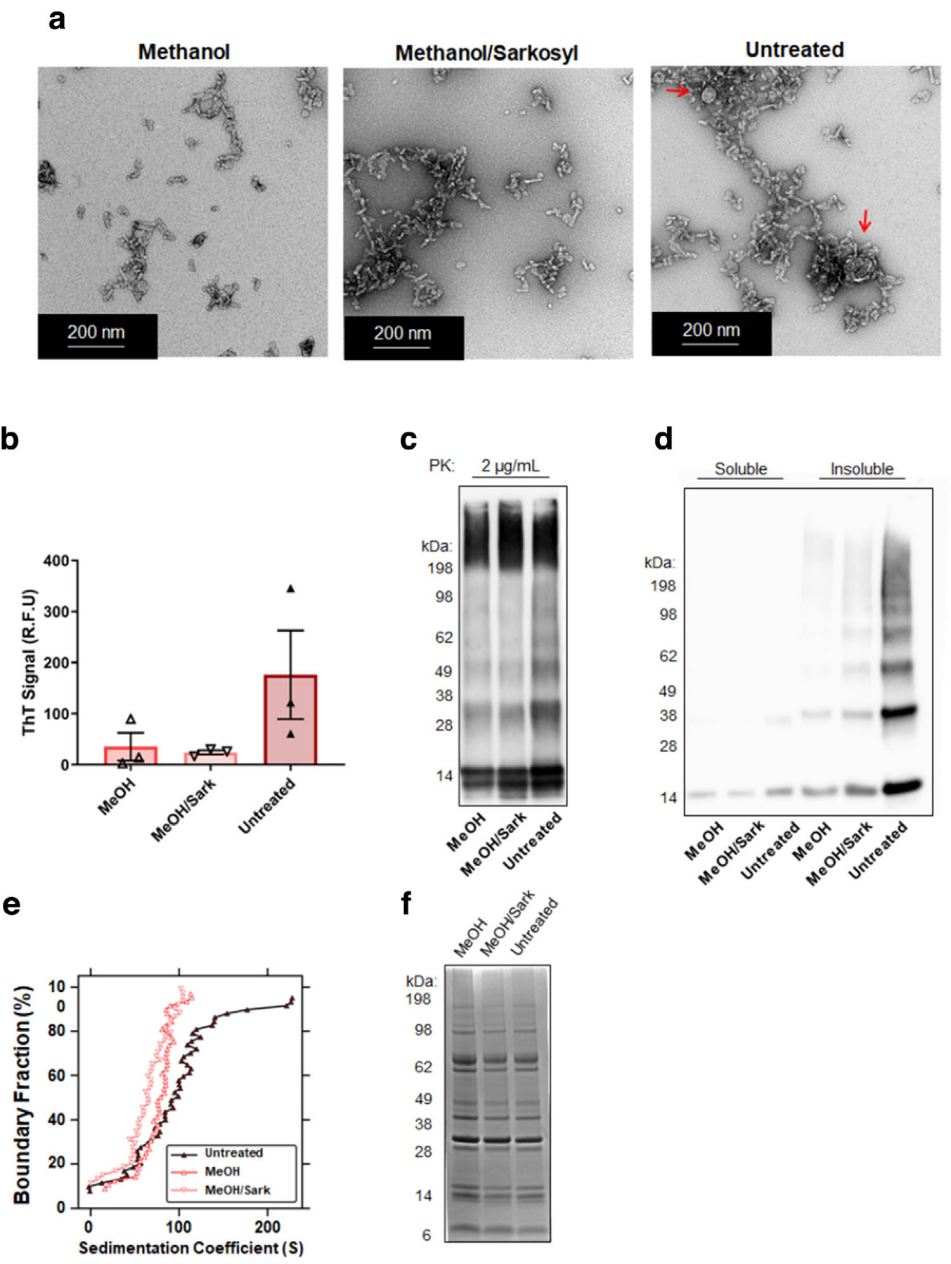
An intact ultrastructure is necessary for EV‐induced modification to αsyn fibrillization. The effect of treating sEVs with methanol alone (MeOH), sarkosyl in addition to methanol (MeOH/Sark), or left untreated (Untreat) to their ability to alter αsyn fibrillization was assessed by: (a) TEM, where red arrors indicate the detection of intact sEVs following PMCA in the untreated group, (b) ThT on protein exposed to PMCA for 12 h, where values obtained for each experimental replicate represented the average fluorescence of triplicate wells after subtraction of a blank well to account for background fluorescence (R.F.U = relative fluorescent units). Data presented as mean±SEM (*n* = 3), (c) western immunoblot in the presence of PK, (d) western immunoblot on samples separated to soluble and insoluble fractions and (e) analytical centrifugation. Shown are enhanced van Holde‐Weischet integral distributions from extrapolation of sedimentation velocity data displayed in Fig. S1D–F. Apparent sedimentation coefficient distributions are plotted as a function of boundary fraction for αsyn in the presence of untreated EVs (upright black triangles, red fill), MeOH‐treated EVs (upright coral red triangles, white fill), or MeOH/Sark‐treated EVs (inverted light‐coral red triangles, white fill). (f) Coomassie of untreated sEVs and those chemically modified with methanol alone or methanol or sarkosyl

The modified sEVs were then added to αsyn and exposed to PMCA. The morphology of αsyn products was first assessed using TEM. Similar to Figure 3, intact sEVs were observed in the untreated sEV group (indicated by red arrows). No vesicles were identified in either of the other groups and no overt differences were observed in the morphology of the αsyn products between the groups (Figure 6a). In order to determine the ability of sEVs to alter the rate of fibrillization, ThT fluorescence was measured at one timepoint (12 h) which was selected to represent the early rises in ThT fluorescence observed in previous ThT experiments (Figure 1e and 4a). Compared to the ThT fluorescence exhibited in the presence of untreated control sEVs, methanol alone (MeOH) and methanol and sarkosyl (MeOH/Sark) treated sEVs exhibited a decreased ThT signal (Figure 6b; MeOH, MeOH/Sark and untreat: 35.3 ± 27.2, 24 ± 4.0 and 176.1 ± 86.6 R.F.U, respectively). The western immunoblot techniques testing the degree of protease resistance and insoluble load was next employed on samples exposed to PMCA for 72 h. The PK digestion western immunoblot revealed minor increases in the abundance of species resistant to 2 μg/ml PK in αsyn misfolded in the presence of untreated sEVs compared to methanol and methanol/sarkosyl treated counterparts (Figure 6c). Following separation of soluble and insoluble fractions, low abundances of monomeric material was identified in the soluble fraction of all samples exposed to PMCA, however in the insoluble fraction a greater amount of αsyn product was found in samples containing untreated sEVs compared to both treatment groups (Figure 6d). These findings suggest that chemically modified sEVs have both a reduced ability to accelerate αsyn misfolding and produce less large fibrils. To provide further insight into the size of species modified in this experiment, analytical centrifugation was performed. The raw absorbance‐detected sedimentation profiles are shown in Figure S1D‐F. These data were analyzed by the vHW method as for the previous sEV experiment using intact, untreated vesicles (Figure 4f). Shown in Figure 6e, compared to untreated sEVs, the addition of MeOH‐treated or MeOH/Sark‐treated sEVs resulted in a marked shift in the sedimentation coefficient distribution towards lower *s* value components comparable to αsyn in the absence of sEVs (Figure 6e). This effect appears marginally more pronounced with the addition of sEVs treated by a combination of MeOH/Sark (Figure 6e). Consistent with the ThT and western immunoblot data, these findings suggest that the loss of sEV membrane structure ablates the effects that these particles have on αsyn fibrillization. Finally, a consideration to the effects seen in Figure 6B‐E is whether these differences may be related to changes in the protein profiles between treatment groups. In order to determine whether these treatments caused overt changes to the protein cargo of the sEV, extracts of each treatment group were lysed, resolved on SDS‐PAGE and Coomassie brilliant blue staining performed. The detection of similar profiles and total protein amounts observed between the samples confirmed that the chemical treatments did not cause major changes to the protein composition of sEVs (Figure 6f).

## 4 DISCUSSION

The misfolding and organized aggregation of αsyn in the CNS is a defining feature of the synucleinopathies. Numerous data show misfolded αsyn can contribute to disease pathogenesis (Ugalde et al., 2016), however the molecular triggers which underlie αsyn misfolding in sporadic disease are poorly elucidated. Insight into the events that cause and/or facilitate protein misfolding will be essential to further our understanding into how these diseases develop and identify therapeutic strategies to treat disease.

This work supports a role of EV membranes in facilitating αsyn misfolding in human disease. This was determined using sEVs, biologically‐derived lipid‐rich vesicles, which were found to cause dramatic changes to how αsyn misfolds. This is the first study to thoroughly characterise the effect sEVs have on the formation of misfolded αsyn and was performed using multiple technical approaches to enable strong experiment conclusions to be drawn from the data obtained. Here, sEV‐induced changes included an acceleration of protein misfolding and production of a higher abundance of large species that exhibits enhanced resistance to PK and insoluble protein load. These data were complemented by analytical centrifugation studies that confirmed the addition of sEVs to αsyn significantly increases the sedimentation rate indicative of the formation of larger components. As an assay that requires very little manipulation of the starting material, the analytical centrifugation results are strong evidence for sEVs contributing to the pathogenic properties of misfolded αsyn. Finally, the demonstration that chemically modified sEVs, that have similar protein profiles, lost their ability to alter αsyn fibrillization confirms that it is a component of the lipid membrane that is indispensable for this effect.

It is well established that αsyn interacts strongly with lipids (Ugalde et al., 2019), and as such lipids are the likely candidate driving the effects on αsyn misfolding reported in the current study. Indeed, the lipid‐rich nature of the EV membrane makes them a useful tool to study protein:lipid interactions and provide significant benefits over studies using preparations of synthetic lipids. Lipids are complex molecules and the packaging of phospholipids into biological membranes is highly dependent on their location of production and interactions with other lipids and soluble proteins (van Meer, Voelker, & Feigenson, 2008). This reflects limitations with studying protein:lipid interactions using synthetic lipids which are unable to totally replicate the exact lipid compositions of cell‐derived membranes. For example, preparations of synthetic membrane structures may impose artefacts associated with membrane curvature given that αsyn has been shown to exhibit a strong affinity for highly curved membranes (Kjaer, Giehm, Heimburg, & Otzen, 2009; Middleton & Rhoades 2010; Pranke et al., 2011; Rhoades, Ramlall, Webb, & Eliezer, 2006). As such, methods of preparation using synthetic vesicles or bilayers may show affinities between αsyn and lipids that would not naturally occur within a cell. However, while there are clear advantages to using biologically‐derived sEVs to study αsyn:lipid interactions, there are experimental considerations to the conclusions of the current study. Given the aforementioned notion of membrane curvature as a relevant aspect of αsyn:lipid interactions, the current study does not distinguish whether the loss of activity of sEVs is due to an altered lipid environment and/or a loss of membrane curvature. Additionally, it is also possible that membrane‐associated proteins act as co‐factors and contribute to the interaction. In this regard, even though the Coomassie brilliant blue staining observed in Figure 6f revealed similar profiles between the untreated and chemically modified sEVs, we cannot exclude the possibility of subtle protein changes contributing to the effects seen. Such notions may be addressed in future work, ideally using pure preparations of EVs such as exosomes, microvesicles or apoptotic bodies to test the relevance of EV size and origin on modulating αsyn misfolding. Such investigations leveraged from work of the current study will provide important information on the nuances relevant to how αsyn associates with biological vesicles.

While this work suggests that sEVs may contribute to αsyn misfolding, a consideration to the biological relevance of sEVs to αsyn misfolding is the apparent differences in cellular location, where while αsyn has a high endogenous expression at the synapse (Jakes et al., 1994; Murphy et al., 2000), sEVs are not reported to be localized in this area. For example, one sub‐type of sEVs, exosomes, are produced within the endosomal system and are released into the extracellular space. However both monomeric and disease‐associated αsyn have been found within sEVs (Emmanouilidou et al., 2010) and the packaging of αsyn within sEVs is increased when cells experience lysosome dysfunction (Alvarez‐Erviti et al., 2011; Danzer et al., 2012). Hence, it is possible in a cell that harbours misfolded protein, the cellular response machinery acting to remove aggregates in disease may contribute to αsyn:EV interactions. In addition to intracellular interactions, αsyn may also associate with sEVs in the extracellular space. Small EVs have been isolated from various biological fluids including blood (Caby, Lankar, Vincendeau‐Scherrer, Raposo, & Bonnerot, 2005), urine (Pisitkun, Shen, & Knepper, 2004) and cerebral spinal fluid (CSF) (Vella, Greenwood, Cappai, Scheerlinck, & Hill, 2008) and given that disease‐associated αsyn has been found elevated in the CSF (Unterberger et al., 2014) and plasma (El‐Agnaf et al., 2006) of patients with synucleinopathy disorders, the two are likely to exist in close proximity in the extracellular space in advanced disease.

In conclusion this work has demonstrated that sEVs are powerful modulators of αsyn fibrillization and that the membrane of sEV is indispensable for this effect. Given that they can both accelerate αsyn misfolding and produce a large quantity of large fibrils, this work provides evidence that sEVs may be important contributors to the generation of disease‐associated, pathogenic αsyn in the synucleinopathy disorders.

## CONFLICTS OF INTEREST

The authors declare that they have no conflicts of interest with the contents of this article.

## Supporting information

Figure S1. Raw sedimentation velocity data. Data were used to obtain enhanced vHW distributions shown in Fig. 4F (A–C) and Fig 7E (D–F). (A) αsyn prepared in the presence of untreated sEVs, (B) untreated sEVs alone, (C) αsyn prepared in the absence of untreated sEVs, and αsyn prepared in the presence of either (D) untreated sEVs, (E) MeOH‐treated sEVs, or (F) MeOH/Sark‐treated sEVs. For visual clarity, only every second scan is shown.
