# Supplementary figures and images for: An intact membrane is essential for small extracellular vesicle‐induced modulation of α‐synuclein fibrillization
Source: J Extracell Vesicles. 2020 Dec 10;10(2):e12034. doi: 10.1002/jev2.12034 (PMC7726797; doi:10.1002/jev2.12034)

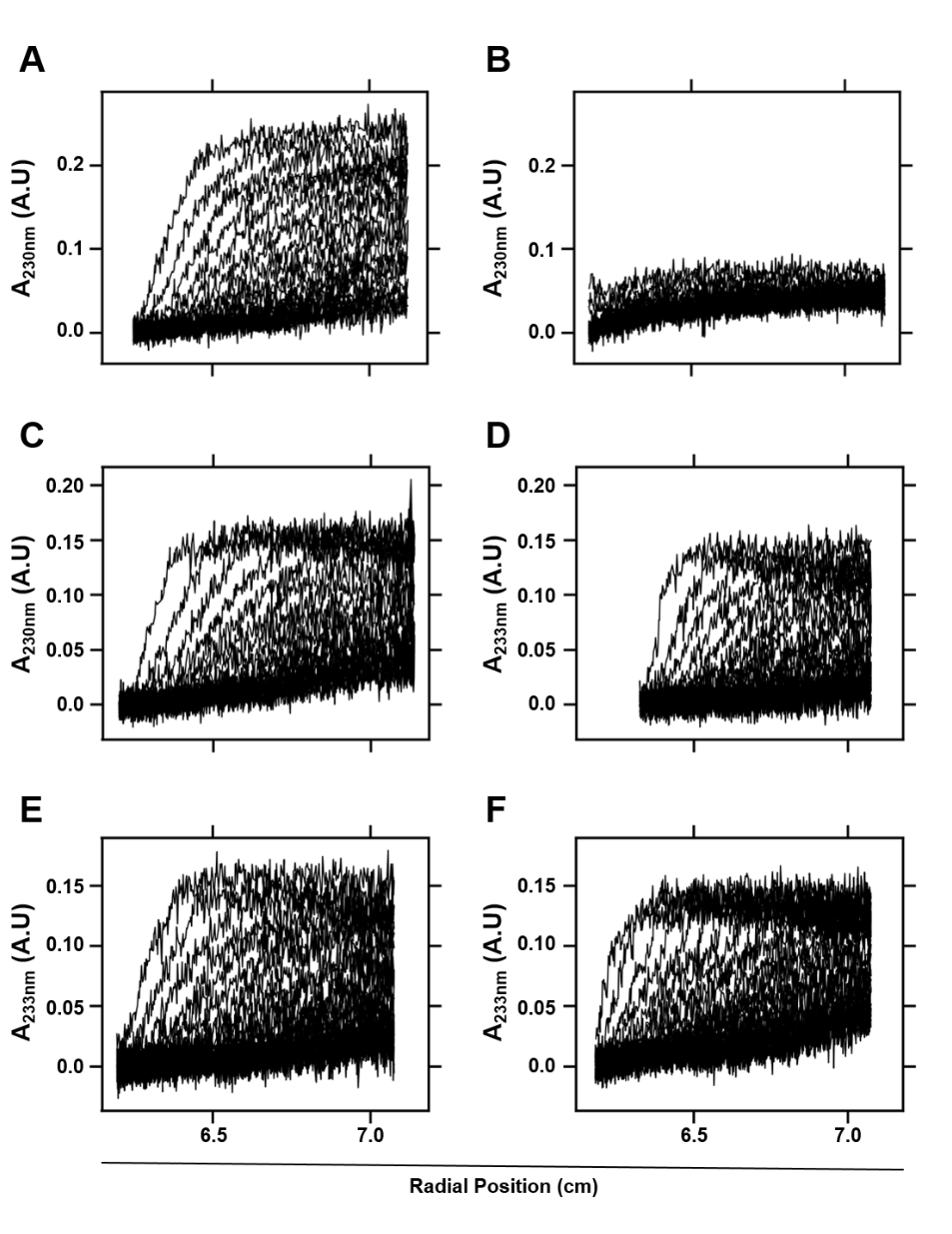

Supplement: Supplementary file 1 — Figure S1. Raw sedimentation velocity data. Data were used to obtain enhanced vHW distributions shown in Fig. 4F (A–C) and Fig 7E (D–F). (A) αsyn prepared in the presence of untreated sEVs, (B) untreated sEVs alone, (C) αsyn prepared in the absence of untreated sEVs, and αsyn prepared in the presence of either (D) untreated sEVs, (E) MeOH‐treated sEVs, or (F) MeOH/Sark‐treated sEVs. For visual clarity, only every second scan is shown. [file JEV2-10-e12034-s001.png]
